# Missing In-Center Hemodialysis Sessions among Patients with End Stage Renal Disease in Banda Aceh, Indonesia

**DOI:** 10.3390/ijerph18179215

**Published:** 2021-08-31

**Authors:** Michael Wei-Chih Liu, Maimun Syukri, Abdullah Abdullah, Li-Yin Chien

**Affiliations:** 1 International Health Program, National Yang Ming Chiao Tung University, Yang-Ming Campus, Taipei 112304, Taiwan; wcliu.ym@gmail.com; 2 Institute of Public Health, National Yang Ming Chiao Tung University, Yang-Ming Campus, Taipei 112304, Taiwan; 3 Division of Kidney and Hypertension, Department of Internal Medicine, Faculty of Medicine, Universitas Syiah Kuala, Banda Aceh 23111, Aceh, Indonesia; maimun_62@yahoo.com (M.S.); abdullah_sawang@yahoo.co.id (A.A.); 4 Division of Kidney and Hypertension, Department of Internal Medicine, Dr. Zainoel Abidin Hospital, Banda Aceh 24415, Aceh, Indonesia; 5 Institute of Community Health Care, College of Nursing, National Yang Ming Chiao Tung University, Yang-Ming Campus, Taipei 112304, Taiwan

**Keywords:** adherence, hemodialysis, kidney-related health literacy, self-care

## Abstract

Indonesian universal health coverage was implemented in 2013 and hemodialysis services became universally accessible, yet few studies have examined patient adherence to hemodialysis schedules. We examined the rates of missed in-center hemodialysis sessions in Banda Aceh and the factors associated with non-attendance. This cross-sectional questionnaire survey included 193 patients receiving in-center hemodialysis. Approximately 28% of the patients missed ≥ 1 hemodialysis session in the month prior to the questionnaire’s administration. About 65% reported attending religious activities as the reason for missing hemodialysis. The level of health literacy was generally low with a mean score of 14.38 out of 26 (55.3%). Multivariate logistic regression analyses showed that patients with educational levels higher than elementary school were less likely to miss hemodialysis sessions. Participants who performed more self-care behaviors had lower odds of missing hemodialysis sessions. Every unit increase in the health literacy score was associated with increased odds of missing hemodialysis sessions. Emphasizing the importance of attending hemodialysis sessions and modifying hemodialysis schedules based on patients’ needs is essential. Patients who miss hemodialysis sessions should be reminded of all self-care behaviors. Health literacy among hemodialysis patients should be improved, with emphasis on patient safety, advanced knowledge, and critical health literacy.

## 1. Introduction

Indonesia, like other developing countries, is experiencing a rise in noncommunicable diseases [1], including chronic kidney disease (CKD) and end-stage renal disease (ESRD); the rise in these diseases is associated with the increasing number of older people and the rapid urbanization that accompanies economic growth. The Indonesian Renal Registry indicates that the number of incident cases of ESRD increased from 9649 to 30,831 and the number of prevalent cases rose from 11,484 to 77,892, from 2010 to 2017. Additionally, CKD progression is often neglected, because patient awareness and access to laboratory services are lacking [2]. In-center hemodialysis (HD) is the most common dialysis, and over 98% of Indonesian patients with ESRD receive in-center HD [3].

In Indonesia, treatment of kidney disease had the second highest healthcare cost in 2015, and CKD is the second leading cause of morbidity after heart disease [4]. The Indonesian health reforms [5] led to universal health insurance, which is available for HD, and the coverage comprises two HD sessions per week for 3–4 h per session as standard, and the necessary medications are free. Kidney transplantation for patients with CKD remains scarce, and among the treatments for CKD, attending HD sessions is the most onerous, but HD is essential because it sustains life [6]. Attendance at HD sessions is problematic, and absences from HD sessions are associated with increased risks of mortality, interdialytic weight gain, and elevated phosphate levels [7].

The findings from a literature review by Victoria et al. [8] showed that older age and a higher educational level were associated with better adherence to treatment by patients with ESRD. Patients with spouses and family support reported better adherence to treatment than those who were unmarried, widowed, or divorced. The transportation time to HD centers is associated with higher mortality levels, and it negatively affects quality of life [9]. In addition, longer transportation times could impede adherence to HD schedules [10].

Patients with ESRD must be health literate, because their treatment regimens are complex [11]. Green et al. showed that between 7% and 37% of patients on HD had low health literacy levels [12], and that a lower level of educational achievement independently predicted lower health literacy levels and reduced adherence to treatment regimens. Another study’s findings showed that low health literacy levels were independently associated with missing HD sessions [13].

Self-care behaviors are important for preventing complications among patients undergoing HD [14]. In addition, patients become more confident when they practice self-care and adopt strategies that engage them in a wide range of lifestyle modifications that affect them psychologically and socially [15]. When complete adherence was compared with other levels of adherence to treatment, patients with a shorter dialysis vintage were more likely to miss HD sessions [16].

Banda Aceh is the capital of and the largest city in Aceh Province, the westernmost province of Indonesia. Of the total 967 dialysis centers in Indonesia, 16 are in Aceh. After national health insurance was implemented in 2013, HD services became universally accessible. However, despite significant improvements in the availability and affordability of HD services, the status of patients with ESRD who receive HD has not been assessed for many years in Indonesia. To our knowledge, few studies have examined compliance with HD schedules in Aceh. This study’s objectives were to examine the rate at which patients with ESRD miss in-center HD sessions in Banda Aceh and the factors associated with these patients missing their HD sessions, including their sociodemographic characteristics, transportation time, kidney-related health literacy, self-care behaviors, and dialysis vintage.

## 2. Materials and Methods

### 2.1. Study Design

This was a cross-sectional questionnaire survey.

### 2.2. Sample and Setting

The study participants were recruited at the largest tertiary teaching hospital in Banda Aceh, which has around 770 beds in total. Of the four dialysis centers in Banda Aceh, the dialysis center at this hospital is the largest and has 45 HD machines/beds. The patients on HD in this hospital are estimated to represent over 70% of the patients on HD in Banda Aceh. In-center HD sessions were arranged during morning and afternoon shifts, and the patients underwent 2 HD sessions per week. The inclusion criteria were patients who had received HD for > 3 months and who were aged > 18 years. We excluded patients in wards and emergency departments and those with unstable health conditions. After screening the patients using these criteria, 214 patients were eligible for enrollment, and 21 patients (10%) refused to participate in the study. The remaining 193 patients gave their consent and completed the survey. Universitas Syiah Kuala’s institutional review board approved this study (No. 53/KE/FK/2017).

### 2.3. Data Collection

Patients were approached during their HD sessions, and the data were collected during face-to-face interviews using a structured questionnaire. The structured questionnaire was reviewed by two nephrologists, three resident doctors, and two nurses to ensure its relevance to the local context. A pilot study involving six patients was conducted to ensure that the patients understood the questions. The interviewers were medical students who had received a two-hour training. The interviews were conducted within the first 90 min of an HD session once the patients had relaxed. All interview data were collected and keyed in by the trained interviewers. The data were collected from November 2017 to November 2018.

### 2.4. Measures

The dependent variable, namely missed in-center HD sessions, was evaluated using the question adopted from the ESRD-adherence questionnaire (ESRD-AQ) [17], “During the past month, how many dialysis treatments did you miss completely?” The response categories were “0,” “1,” “2,” “3,” and “4 or more”; the responses were recategorized for a dichotomous data analysis, because very few of the patients’ responses were in the latter categories. The patients were asked other questions from the ESRD-AQ that were related to HD, as follows: “Is your dialysis schedule convenient for you?”; “When was the last time a health professional talked to you about the importance of not missing your dialysis treatment?”; “How important do you think it is to follow your dialysis schedule?”; and “What was the main reason you missed your dialysis treatment last month?”. The independent variables included patients’ sociodemographic factors, namely age, sex, marital status, education level, employment status, income, out-of-pocket medical expenses, place of residence, namely urban or rural, and their transportation time; kidney-related health literacy level; self-care behaviors; and dialysis vintage.

To evaluate a patient’s kidney-related health literacy level, we adopted an established reliable and valid instrument that was developed by Shi et al. [18]. This instrument contains 26 multiple-choice questions, and it investigates ESRD-specific literacy through seven subscales, as follows: the functional literacy subscale (five items) asked patients about the purpose of dialysis and associated treatments, care of the arteriovenous fistula, blood pressure control, and uremia; the basic health knowledge literacy subscale (four items) inquired about calcium, potassium, and phosphorous intakes, foods to avoid, medical reasons for monitoring fluid intakes, and fluid consumption; the communication literacy subscale (four items) asked patients about responses when they were uncertain about medical advice, prescribed medicines, laboratory results, or feeling unwell after taking medication; the interactive literacy subscale (three items) challenged patients about receiving treatment advice from nonmedical sources and encountering problems with dialysis; the advanced health knowledge literacy subscale (five items) examined a patient’s knowledge about common complications, kidney transplantation, comparisons to peritoneal dialysis, and precautions when taking oral medication; the critical literacy subscale (three items) checked whether patients knew their allowable weight gain, could calculate their ideal daily water intake, and knew about self-care; the patient safety literacy subscale (two items) inquired about fall and infection prevention. Each correct answer scored 1 point, and the total score ranged from 0 to 26, with a higher score indicating a higher level of kidney-related health literacy.

We developed a set of questions that asked patients how they managed self-care at home that required “yes” or “no” responses. The questions included “visit the clinic regularly”, “keep diet under control”, “maintain blood sugar level”, “maintain blood pressure level”, “measure blood pressure at least once per week”, “record blood pressure regularly”, and “maintain weight”. The internal consistency of the whole self-care scale was assessed using the Kuder-Richardson Formula 20, and it was 0.62.

We asked the patients about their blood test results; however, the patients did not keep the test results and were unaware of the values. Therefore, after obtaining the patient’s consent, we turned to the hospital laboratory department for the participants’ laboratory data including blood urea nitrogen (BUN), creatinine, potassium, and calcium levels. Patient’s name and chart number were used to find the test results. The most recent available test results were used. However, the test results were available for only 92 (47.67%) out of the 193 patients through the system. The data were missing due to the use of different patient names (confusion between formal name, informal name, and nick name), unmatched chart numbers, and problems in the computerized system.

### 2.5. Data Analysis

The statistical analyses were conducted using the Stata Statistical Software (Release 15). Regarding the descriptive statistics, the continuous variables were presented as means with their standard deviations (SDs), and the categorical variables were presented as numbers and percentages. Pearson’s chi-squared test or Fisher’s exact test was used to compare the proportions in the bivariate analysis. Analyses of variance or Student’s t-test was used to compare the means. The level of significance was set at 0.05. Backward selection procedures with restrictive criteria (*p* < 0.1) were used to select multiple predictors in the binary multivariate logistic regression models [19,20,21].

## 3. Results

### 3.1. Sociodemographics

The patients’ mean age was 51.12 (11.02) years (range: 19–77 years). Most of the study participants (67.36%) were aged between 40 years and 60 years. Over 60% of the participants were men, and over 50% of the participants were unemployed. Approximately 25% had an elementary school education level or lower. Around 50% of the participants were on low income according to the cutoff point in the Indonesia demographic survey [22]. Over 90% of the patients spent < 10% of their income on out-of-pocket medical expenses. Approximately 60% of the patients lived in rural areas (Table 1).

### 3.2. Hemodialysis-Related Characteristics

Regarding the binary outcome, 27.99% (*n* = 54) of the participants had missed ≥ 1 HD session entirely during the previous month, and they comprised the miss group. Of the participants in the miss group, 75.93% (*n* = 41) had missed 1 session, 12.96% (*n* = 7) had missed 2, 3.70% (*n* = 2) had missed 3, and 7.41% (*n* = 4) had missed ≥ 4 HD sessions.

Regarding the main reasons for missing HD sessions, 64.81% (*n* = 35) reported that they had other things to do, including attending events that mostly comprised religious activities or religious holidays. Furthermore, 7.41% (*n* = 4) reported that they had transportation problems and 7.41% (*n* = 4) reported they did not want to or could not undergo HD, because they felt physically unwell or felt healthy and, therefore, did not need HD, or they just wanted to know what would happen. The remaining reasons for not attending HD sessions included vascular access thrombosis, hospitalization, and forgetfulness.

Over 90% of the patients thought their HD schedules were convenient, while the remaining patients considered the schedules inconvenient, because they were too early (2.07%), were too late (0.52%), conflicted with work schedules (2.07%), conflicted with medication times (0.52%), or for other reasons (3.63%). Regarding the last time a health professional talked to the participants about the importance of not missing HD sessions, 60.10% of the participants reported that this had occurred during their first HD session or never, 23.32% reported this had occurred “this week or last week,” and 16.58% reported this had occurred “last month or more than 1 month ago.” Regarding the patients’ perceptions about the importance of following their HD schedules, only 2.59% reported it was “a little important”.

The mean transportation time and dialysis vintage were 38.76 (SD 49.42) min and 2.98 (1.66) years, respectively. Transportation time and dialysis vintage were not significantly different between the miss and no miss groups (mean transportation time: 45.83 (53.86) versus 36.01 (47.51), respectively, *p* = 0.23; mean dialysis vintage: 2.93 (1.80) versus 3 (1.60), respectively, *p* = 0.81).

### 3.3. Kidney-Related Health Literacy

Since the number of items in the health literacy subscales differed, the mean score was divided by the full score to yield a percentage that could be compared across subscales. The mean (SD) score for kidney-related health literacy was 14.38 (3.27) out of a full score of 26, yielding a percentage of 55.31% (14.38/26). The scores on the seven health literacy subscales were as follows: interactive literacy: 2.28/3 (76.0%); basic health knowledge: 2.76/4 (69%); communication literacy: 2.66/4 (66.5%); functional literacy: 2.61/5 (52.2%); patient safety: 0.97/2 (48.5%); advanced health knowledge: 2.01/5 (40.2%); and critical literacy: 1.08/3 (36.0%). Except for the advanced health knowledge score, the patients who missed HD sessions had higher mean scores on all the subscales than those who did not miss HD sessions. Compared with the no miss group, the miss group had significantly higher scores on the basic health knowledge, interactive literacy, and patient safety subscales (Table 2).

### 3.4. Self-Care Behaviors

Regarding the seven self-care behaviors, the rates were 78.76% for checking blood pressure at least once a week, 75.13% for regular diet control, 59.59% for maintaining blood pressure, 54.92% for visiting clinics regularly, 44.56% for maintaining weight, 43.52% for maintaining blood sugar levels, and 24.35% for recording blood pressure. Patients who missed HD sessions had a lower rate of practicing self-care behaviors than those who did not miss HD sessions, and the differences were significant for regular clinic visits (40.74% versus 60.43%, *p* = 0.01), maintaining blood sugar levels (25.93% versus 50.36%, *p* = 0.002), checking blood pressure (68.52% versus 82.73%, *p* = 0.03), and maintaining weight (31.48% versus 49.64%, *p* = 0.023) (Table 3).

### 3.5. Blood Test Results

The test results were available for only 92 (47.67%) out of the 193 patients. The blood test results are presented in Table 4. Out of the 92 patients, about 78% had high creatinine, 73% had either high or low calcium, 70% had high BUN, and 58% had either high or low potassium levels. There were no significant differences in the test values between those who missed HD sessions and those who did not.

### 3.6. Factors Associated with Missing Hemodialysis Sessions

Table 5 presents the final models from the multivariable logistic regression analyses. Other included variables (as in the Measures) were not significantly related to missing HD sessions and were thus dropped from the model. Model 1 used the total kidney-related health literacy and total self-care behavior scores as predictors. Model 2 used the individual kidney-related health literacy subscale and individual self-care behavior as predictors. When the total scores were considered, kidney-related health literacy and self-care behaviors were significantly associated with missing HD sessions. Each one-point increase in kidney-related health literacy was associated with an increased risk of missing HD sessions (odds ratio [OR] = 1.19, 95% confidence interval [CI] = 1.05–1.34). Each additional self-care behavior component was associated with a reduced risk of missing HD sessions (OR = 0.85, 95% CI = 0.74–0.97). An educational level that was higher than elementary school (OR = 0.43, 95% CI = 0.2–0.93) was associated with reduced odds of missing HD.

In model 2, basic health knowledge literacy (OR = 1.84, 95% CI = 1.17–2.89) and interactive literacy (OR = 1.74, 95% CI = 1.06–2.84) were associated with increased odds of missing HD sessions. Blood pressure checks (OR = 0.33, 95% CI = 0.15–0.75) and maintaining blood sugar levels (OR = 0.42, 95% CI = 0.20–0.87) were associated with reduced odds of missing HD sessions. An educational level that was higher than elementary school (OR = 0.36, 95% CI = 0.19–0.84) was associated with reduced odds of missing HD sessions.

## 4. Discussion

We found that 28% of the patients had missed HD sessions in the month that preceded the administration of the questionnaire. A previous review reported that the missed HD session rate varied from 0% to 35%, but the methods used to determine these rates differed across the studies analyzed [23]. For example, some studies defined missed HD as skipping HD entirely, while others applied a cutoff value for the percent reduction in the prescribed HD dosage. Furthermore, the findings from a study in Aceh Utara, which is on the outskirts of Aceh, showed that based on attendance for 3 months before the study, 40% of the 110 study participants did not adhere to their HD schedules [24]. However, while the participants’ age and sex distribution in the current study and in the study in Aceh Utara appear similar, differences exist regarding the time frames in which the missed HD sessions were evaluated; therefore, the non-adherence rates are not comparable.

In this study, patients who missed HD sessions stated that they were not contacted regarding their missed session and just underwent HD at the next scheduled time. Missing HD sessions can cause cardiovascular complications and hospitalization and can increase mortality [16]. Cohen et al. [25] suggested rescheduling HD on the subsequent day when patients missed sessions. Although the risks associated with higher hospitalization rates and emergency room visits remained, the magnitude of the elevations in risk appeared to be less pronounced for patients who missed a few HD sessions compared with those who missed many HD sessions. However, in real practice, compensation for a missed HD dosage rarely occurs, because patients are reluctant to attend HD sessions on consecutive days, especially when additional effort is required to travel to the HD center. Moreover, HD centers may not have the capacity to permit rescheduling, because of equipment and personnel constraints. However, patients are advised that emergency services are in place if they have urgent needs. Based on the patients’ reports, we found that the in-center health professionals rarely reiterated the importance of adhering to HD schedules to patients, and that over 60% of the patients had never been advised or had only been advised of this during their first HD session. Therefore, patients should be encouraged to adhere to their HD schedules, and the consequences of missing HD sessions, including safety concerns and potential complications, should be emphasized to patients and their families repeatedly [7]. HD center staff should assess patients for compensatory HD and if necessary, reschedule and provide compensatory HD.

“Having other things to do” was the most frequently reported reason for missing HD. Further exploration indicated that most patients missed HD because they were attending religious events or were away for religious holidays. According to the 2010 census [26], over 98% of the population in Aceh is Muslim. Aceh is a conservative religious territory and the only Indonesian province in which residents practice Sharia law officially. Therefore, some patients choose to attend religious activities rather than medical appointments, and they may not be aware of the importance of HD or understand the consequences of missing HD [27]. A previous study’s findings showed that religiosity was unrelated to adherence to medical care [28]. On the contrary, another study’s findings showed that religiosity was positively associated with HD adherence [29]. Therefore, more studies are needed that examine the role of religiosity in HD adherence. Nonetheless, planning and rescheduling HD sessions with hospital staff may help mitigate the health impact of missing HD.

The finding that some patients did not want to undergo HD because they felt healthy or they wanted to find out what would happen is worrying. This reflects a lack of kidney-related health knowledge [30]. Health professionals should regularly assess and address patients’ misunderstandings. Slightly less than 10% of the study’s participants thought their HD schedules were inconvenient. Home dialysis could be an option for these patients [31], but it is rarely provided in the clinical setting. Advocating home HD or PD could improve patients’ adherence to dialysis, especially if they have conflicting schedules and transportation problems.

The mean kidney-related health literacy score in this study was 14.38 out of a full score of 26 (55.31%), which is lower than that reported in a previous study of patients on HD in Taiwan (19.70/26; 75.77%) [18]. The results showed that level of health literacy was generally low among patients on HD in Banda Aceh. Of the seven subscales of health literacy, interactive literacy (76.0%), basic health knowledge (69%), and communication literacy (66.5%) scored higher than critical literacy (36%), advanced health knowledge (40.2%), patient safety (48.5%), and functional literacy (52.2%). The results seem to suggest that participants were more informed about health literacy regarding knowledge of daily life adjustment required for ESRD and interaction with medical personnel. However, health literacy regarding the importance of dialysis and treatment, prevention of potential complications, and practical applications of care for their own health needs to be improved. The aforementioned results suggest that health literacy among patients on HD should be improved, with emphasis on patient safety, advanced knowledge, and critical health literacy.

Contrary to our expectations, a higher kidney-related health literacy score was associated with a significant increase in the odds of missing HD sessions. This finding differs from previous studies, as they showed that higher health literacy levels were related to higher levels of HD adherence [32]. Of the seven subscales, interactive literacy and basic health knowledge generated the highest mean scores and were positively related to missing HD sessions in the multivariate model. We noted that the mean score difference between those who missed HD and those who did not was only 1.3 (out of the full score of 26). Therefore, a higher score in health literacy may not be sufficient for not missing their HD sessions among a group of patients whose health literacy was in average low. Those who had a higher, but not high enough health literacy score may believe that they know more about their health and want to keep their ordinary life. Therefore, they may attend other activities or events instead of HD sessions. Further studies are needed to examine this assumption and to evaluate a high enough health literacy score that shows a positive effect on adherence to HD sessions. Nonetheless, patients reporting higher health literacy may require more consultation with healthcare professionals on adherence to their HD sessions.

Higher self-care behavior scores were associated with reduced odds of missing HD sessions, which concurs with the findings from previous studies [33]. Self-care behaviors are important for preventing complications among patients with CKD [34]. Of these self-care behaviors, the associations between missing HD sessions and monitoring and maintaining blood pressure and blood sugar levels were the most distinct. Healthcare workers should advocate self-care behaviors among patients who receive HD because this could benefit patient adherence to HD schedules and other treatment regimens, including restricting liquid and dietary intakes, which are important for managing patients with ESRD [35].

Higher educational levels, that is, beyond elementary education, were associated with reduced odds of missing HD in this study. This finding concurs with findings from previous studies that showed that higher educational levels were associated with adherence to HD sessions [36]. Dialysis vintage and transportation time were not significantly associated with missing HD session in the study. The lack of association could be because most participants had a short dialysis vintage and transportation time in this study.

In the 92 patients whose blood test results were available, the high rate of abnormal blood test results suggests a need for further scrutiny, though the test results were not related to missing HD sessions. The potential underlying reasons for deviant values such as under-dosage of HD [37], poor self-care behavior [38], and nutrition problems [39] should be examined in a future study.

This study’s limitations include its cross-sectional design, and the correlations reported do not imply causation. Non-adherence to HD is a complex problem. Additionally, this study did not include other potentially important factors such as environmental, psychological, and clinical factors. Future studies should include these variables and examine the reciprocal relationships between the variables and non-adherence to HD for a more comprehensive understanding. Although this study was conducted at a single HD center in Banda Aceh, it treats > 70% of the patients with ESRD who receive HD in the area. Nonetheless, the results may not reflect non-adherence to HD in patients from smaller cities and rural areas. Though the questionnaires had been reviewed by experts and the interviewers were trained, we did not examine the psychometric properties of the scales and inter-rater reliability. The study variables were derived from patients’ self-reports; hence, we cannot verify the validity of these data. Further, a social desirability bias may be found when adherence behaviors are scrutinized. Moreover, blood test results were not available for more than half of the study participants. Future studies are needed to examine the association between blood test results and non-adherence to HD sessions.

## 5. Conclusions

Over one-quarter of the patients with ESRD missed HD sessions in the month preceding the administration of the questionnaire, which suggests the need to emphasize the importance of adhering to HD schedules. Since the reasons given for missing HD were related to religious activities mainly, rescheduling HD sessions or compensatory HD should be considered. Healthcare professionals could initiate discussions with patients about choices between HD and religious events and provide or suggest alternative solutions, especially to religious patients.

Self-care behaviors were negatively associated with missing HD sessions; therefore, patients who miss HD sessions should be reminded of all self-care behaviors. Self-care behaviors, including monitoring blood sugar and blood pressure levels, should be advocated. We could provide patients and their caregivers with more ESRD- and HD-specific educational materials to increase their self-care skills and motivate them to engage in self-care behaviors.

The patients who reported higher kidney-related health literacy levels were more likely to miss HD sessions, which suggests that better communication with these patients is required. Kidney-related health literacy should be further promoted among patients with ESRD. Healthcare professionals should be encouraged to interact and communicate with patients to correct misconceptions.

## Figures and Tables

**Table 1:** The study participants’ characteristics (*N* = 193).

		Hemodialysis Group						
Characteristics		Overall		Miss Group		No Miss Group		*p*-Value
		*n*	%	*n*	%	*n*	%	
Sex								0.51
	Man	118	61.14	31	57.41	87	62.59	
	Woman	75	38.86	23	42.59	52	37.41	
Marital status								0.57
	Single	18	9.33	4	7.41	14	10.07	
	Married	175	90.67	50	92.59	125	89.93	
Education level								0.03
	Elementary school or less	41	21.24	17	31.48	24	17.27	
	More than elementary school	152	78.76	37	68.52	115	82.73	
Employment								0.26
	No	120	62.18	37	68.52	83	59.71	
	Yes	73	37.82	17	31.48	56	40.29	
Income								0.51
	Low	107	55.44	32	59.26	75	53.96	
	Middle and high	86	44.56	22	40.74	64	46.04	
Out-of-pocket medical expenses (% income)								0.78
	≤10%	176	91.19	50	92.59	126	90.65	
	>10%	17	8.81	4	7.41	13	9.35	
Place of residence								0.90
	Rural	113	58.55	32	59.26	81	58.27	
	Urban	80	41.45	22	40.74	58	41.73	

**Table 2:** Kidney-related health literacy among the study participants.

	Hemodialysis Group						
	Overall		Miss Group		No Miss Group		*p*-Value
	Mean	SD ^1^	Mean	SD ^1^	Mean	SD ^1^	
Kidney-related health literacy							
Total score ^2^	14.38	3.27	15.30	2.24	14.02	3.54	0.003
Functional literacy	2.61	1.15	2.76	1.01	2.55	1.20	0.27
Basic health knowledge	2.76	0.90	3.04	0.73	2.65	0.94	0.003
Communication literacy	2.66	1.14	2.80	1.12	2.61	1.15	0.31
Interactive literacy	2.28	0.81	2.48	0.67	2.20	0.85	0.02
Advanced health knowledge	2.01	1.12	1.89	1.06	2.05	1.15	0.37
Critical literacy	1.08	0.73	1.20	0.76	1.04	0.72	0.15
Patient safety	0.97	0.52	1.13	0.48	0.91	0.53	0.01

^1^ SD, standard deviation. ^2^ The full scores were 26 for total score, 5 for functional literacy, 4 for basic health knowledge, 4 for communication literacy, 3 for interactive literacy, 5 for advanced health knowledge, 3 for critical literacy, and 2 for patient safety.

**Table 3:** Self-care behaviors among the study participants.

		Hemodialysis Group						
Characteristics		Overall		Miss Group		No Miss Group		*p*-Value
		*n*	%	*n*	%	*n*	%	
Regularly visiting the clinic								0.01
	No	87	45.08	32	59.26	55	39.57	
	Yes	106	54.92	22	40.74	84	60.43	
Regular diet control								0.09
	No	48	24.87	18	33.33	30	21.58	
	Yes	145	75.13	36	66.67	109	78.42	
Regularly maintaining blood sugar								0.002
	No	109	56.48	40	74.07	69	49.64	
	Yes	84	43.52	14	25.93	70	50.36	
Maintaining blood pressure								0.30
	No	78	40.41	25	46.30	53	38.13	
	Yes	115	59.59	29	53.70	86	61.87	
Blood pressure checked at least once weekly								0.03
	No	41	21.24	17	31.48	24	17.27	
	Yes	152	78.76	37	68.52	115	82.73	
Recording blood pressure level								0.054
	No	146	75.65	46	85.19	100	71.94	
	Yes	47	24.35	8	14.81	39	28.06	
Regularly maintaining weight								0.02
	No	107	55.44	37	68.52	70	50.36	
	Yes	86	44.56	17	31.48	69	49.64	

**Table 4:** Blood test results among the study participants (*n* = 92).

		Hemodialysis Group						
		Overall		Miss Group		No Miss Group		*p*-Value
		*n*	%	*n*	%	*n*	%	
BUN (mg/dL)								0.65
	≥100	64	69.57	22	66.67	42	71.19	
	others	28	30.43	11	33.33	17	28.81	
Creatinine (mg/dL)								0.34
	≥7	72	78.26	24	72.73	48	81.36	
	others	20	21.74	9	27.27	11	18.64	
Potassium (mEq/L)								0.14
	≥4.7	46	50	16	48.48	30	50.85	
	normal	39	42.39	12	36.36	27	45.76	
	<3.5	7	7.61	5	15.15	2	3.39	
Calcium (mg/dL)								0.11
	≥10	22	23.91	12	36.36	10	16.95	
	normal	25	27.17	8	24.24	17	28.81	
	<8.5	45	48.91	13	39.39	32	54.24	

BUN, blood urea nitrogen.

**Table 5:** Multivariable logistic regression models for factors associated with missing hemodialysis sessions.

	Model 1 ^1^			Model 2 ^2^		
Factors Associated with Missing in-Center Hemodialysis Sessions	Odds Ratio	95% Confidence Interval		Odds Ratio	95% Confidence Interval	
		Lower	Upper		Lower	Upper
**More than elementary school education** **(reference: elementary school or less)**	0.43	0.20	0.93	0.36	0.16	0.81
**Total health literacy score (reference: unit score)**	1.19	1.05	1.34			
**Basic health knowledge** **(reference: unit score)**				1.84	1.17	2.89
**Interactive literacy** **(reference: unit score)**				1.74	1.06	2.84
**Total self-care score** **(reference: unit score)**	0.85	0.74	0.97			
**Blood pressure checks at least once a week** **(reference: no check)**				0.33	0.15	0.75
**Maintain blood sugar level** **(reference: no maintenance)**				0.42	0.20	0.87

^1^ R^2^ = 9.99%, *p* = 0.0001, Akaike Information Criterion = 1.129, Bayesian Information Criterion = −778.178, mean variance inflation factor = 1.03. ^2^ R^2^ = 13.61%, *p* < 0.001, Akaike Information Criterion = 1.133, Bayesian Information Criterion = −761.204, mean variance inflation factor =1.06.

## Data Availability

The data presented in this study are available on request from the corresponding author. The data are not publicly available due to privacy.
